# Two Important Factors in Tuberculosis of the Genito Urinary Tract

**Published:** 1914-11

**Authors:** 


**Two Important Factors in Tuberculosis of the Genito Urinary Tract.** J. Henry Down in "Urologic and Cutaneous Review" sums up tuberculosis of the Genito-urinary tract as follows:

"Tubercle bacilli can rarely be found in the urine, unless there is ulcerative processes present over which the urine flows.

Hemorrhage may occur early, before the bladder becomes involved, and markedly so.

Hemorrhage my occur early, before the bladder becomes involved, but it is rare.

The inflammatory action not only involves the mucus membrane, but the interstitial connective tissue in which latter contraction takes place, thus diminishing caliber.

Dowd makes the statement that the normal bladder will hold 12 to 14 ounces of fluid, with little or no inconvenience; whereas, even in the presence of tuberculosis with no subjective symptoms, 6 to 8 ounces will cause great distress: this does not occur in any simple inflammatory process.

After stating that silver nitrate solutions, even in 1-30 000, causes a varying amount of irritability in simple conditions for 6 or 8 hours, when tuberculosis is present this irritation, but markedly increased, continues from 24 to 36 hours.

Silver should not be used for irrigation of tuberculosis of the bladder, it irritates rather than soothes the trouble.

From all reports it is evident that phosphorus is fast gaining first place as a medicinal remedy in the treatment of general tuberculosis: phosphorus holds a similar place in urinary tuberculosis: surgery, up to date, has been a failure."

